# Determinants of Antenatal Care Utilization Among Reproductive Age Women in Somaliland Using Somaliland Health Demographic Survey 2020 Data

**DOI:** 10.1089/whr.2024.0155

**Published:** 2025-05-21

**Authors:** Hodo Abdikarim, Abdisalam Hassan Muse, Mukhtar Abdi Hassan, Saralees Nadarajah, Yahye Hassan Muse

**Affiliations:** ^1^ Faculty of Science and Humanities, School of Postgraduate Studies and Research (SPGSR), Amoud University, Borama 25263, Somalia.; ^2^ School of Graduate Studies, University of Hargeisa, Hargeisa 25263, Somalia.; ^3^ Department of Mathematics, University of Manchester, Manchester M13 9PL, UK.

**Keywords:** antenatal care, ANC utilization, Somaliland, maternal health, sociodemographic factors, SLDHS 2020

## Abstract

**Background:**

This study aimed to identify the determinants of antenatal care (ANC) utilization among reproductive-aged women in Somaliland. Understanding these factors is crucial for improving maternal health. This study utilized data from the 2020 Somaliland Demographic Health Survey (SLDHS), which encompasses urban, rural, and nomadic residencies across six geographic zones in Somaliland.

**Methods:**

This cross-sectional study included 2741 women aged 15–49 based on data from the SLDHS 2020. The primary outcome variable was ANC utilization, which was measured as a binary variable (utilization vs. nonutilization). Descriptive statistics and binary logistic regression analyses were conducted to identify the factors associated with ANC utilization.

**Results:**

The study found that higher maternal education levels (adjusted odds ratios [OR] = 2.15, 95% confidence interval [CI]: 1.47–3.14), urban residence (adjusted OR = 1.36, 95% CI: 1.09–1.70), higher household wealth quintiles (adjusted OR = 3.45, 95% CI: 2.50–4.76), husband’s education level (adjusted OR = 1.87, 95% CI: 1.36–2.56), and exposure to mass media (adjusted OR = 1.75, 95% CI: 1.32–2.31) were significantly associated with increased ANC utilization.

**Conclusion:**

Education, economic status, and accessibility are the key determinants of ANC service uptake in Somaliland. These findings provide valuable insights for health planners and policymakers aiming to improve ANC utilization and maternal health outcomes in Somaliland.

## Introduction

Antenatal care (ANC), provided by skilled health care professionals, is a specialized service for pregnant women that aims to promote optimal health outcomes for both the mother and child throughout the entire duration of the pregnancy. It is one of the four key components of safe motherhood initiatives that focus on maintaining good health during pregnancy and the early postpartum period.^1^ ANC includes a range of interventions to detect potential risks, manage pregnancy-related conditions, and provide health education.^2^

ANC is a critical approach for minimizing maternal morbidity and mortality by increasing the likelihood of the timely detection of high-risk pregnancies. Although it may not prevent all causes of maternal and neonatal mortality, it plays a vital role in the early recognition and prevention of various preexisting conditions. Furthermore, it serves as a point of entry for the integrated use of skilled health care personnel. Several empirical studies on preventive services have emphasized the importance of regular monitoring of women during pregnancy to decrease birth-related complications, provide supportive care, and promote safer motherhood.^3^

To ensure that ANC services are of the highest quality, they must be initiated before the 12th week of pregnancy and attended to at the recommended intervals throughout the entire gestational period. In 2013, 58.6% of pregnant women globally received ANC before the 12th week of pregnancy, although there were considerable disparities across regions.^4^ Based on a comprehensive evaluation of various ANC models, the World Health Organization (WHO) recommends a minimum of four antenatal visits.^5,6^ The WHO provides thorough guidelines on the content of these visits, which include measuring blood pressure, testing urine for bacteriuria and proteinuria, testing blood for syphilis and severe anemia, and optional weight and height measurements.^7^

ANC utilization varies globally. According to data from 2010 to 2016, 62% of pregnant women worldwide received a minimum of four antenatal visits, as recommended by the WHO. However, there are stark differences in the proportion of women attending ANC visits in different regions. For instance, in New York City, 58% of women received the recommended minimum,^8^ while in Western Jamaica, 53.4% attended seven or more visits and 27.2% attended four to six visits.^9^ In India, only 21% of women received the minimum recommended visits,^10^ whereas in Nepal, 85% had at least one ANC visit.^11^ In Jordan, 63.4% of women receive the recommended minimum (2) visits, while in Niger, 74.4% of women attend at least one ANC visit.^12^ In Nigeria, only 54% of women received the minimum recommended visits,^13^ and in Kenya, only 14% of women attended their first ANC visit in the first trimester, with only 46% making the recommended four visits.^14^ In Ethiopia, 63.77% of women receive a minimum number of recommended visits.^1^

ANC services available to women of reproductive age in Somaliland demonstrate significant heterogeneity, primarily driven by geographic location and socioeconomic disparities. Women in urban areas generally have better access to well-equipped health care facilities and a higher frequency of ANC visits compared to those in rural or nomadic areas, where access is often limited by distance and inadequate infrastructure. Furthermore, service availability is influenced by household wealth, with wealthier women more likely to afford transportation, out-of-pocket expenses, and private health care options. In addition, variations in health care provider training and resources contribute to differing levels of service quality, making it crucial to address these disparities through policies that promote equitable health care distribution and support for underserved populations. This multifaceted landscape necessitates targeted interventions that improve both the availability and quality of ANC services across Somaliland, especially in marginalized areas.^4,15–18^

In a recent review of low- and middle-income countries, Banke-Thomas OE emphasized that the education of the mother and her partner was the most significant factor affecting the utilization of maternal health care services.^19^ Additional studies conducted in various regions of the world have found that the use of ANC is influenced by the mother’s age, parity, interaction with health care providers, and the cost of ANC care, as well as factors such as mass media, higher parity, long distance to the health facility, seeking permission to use focused ANC, having an unemployed husband/partner with low-birth-weight babies, and delivery without induction of labor.

Insufficient utilization of antenatal services has also been found to be associated with various factors. In several sub-Saharan African countries, the utilization of ANC services is closely linked to client satisfaction. Numerous studies conducted in Ethiopia have demonstrated that the level of satisfaction among pregnant women with ANC services ranged from 33.4% to 83.9%. For instance, in Jimma town, the satisfaction level was 60.4%; in the Bahir Dar Special Zone, it was 53.3%; in Bursa Woreda of Sidama Zone, it was 33.4%; and in Alganesh Health Center Shire, North West Tigray, it was 83.9%.^1,19,20^

In Somaliland, ANC utilization remains suboptimal, with only 42% of pregnant women receiving ANC services, below the WHO’s recommended minimum of four visits. Utilization rates vary across regions, from 40% in Maroodijeex/Sahil to 19% in Sanaag.^15^ This low uptake highlights the need to explore the determinants of ANC utilization within the local context. While previous studies in low-resource settings have examined barriers to ANC, few have focused on Somaliland’s unique sociocultural and geographic challenges.^1^

This study serves as a starting point for further research that may build upon these findings and explore changes or trends in ANC utilization in subsequent years.

## Materials and Methods

### Study area

The present study was conducted in Somaliland, a country situated in the East African region. Somaliland’s geographical location is characterized by its proximity to Djibouti to the northwest, Ethiopia to the southwest, Somalia to the east, and the Gulf of Aden to the north. The country has a total area of 176,119.2 km^2^, and the country experiences a combination of wet and dry climatic conditions. Somaliland is divided into six geopolitical regions: Awdal, Marodijeh, Sahil, Togdheer, Sanaag, and Sool. The estimated population of the country is 6.2 million, primarily composed of Somali ethnic groups and Muslims. Although the country has experienced some economic growth since its declaration of independence, Somaliland is still a nation with slow economic progress. The lack of international recognition as an independent state has hindered investment and international aid in the region. The majority of the population, both in rural and urban areas, relies on livestock products for their livelihood.

A study analyzed school dropout rates in Somaliland using data from the 2022 National Education Accessibility Survey, which encompassed 1957 households across diverse districts, such as Hargeisa, Burao, and Berbera, allowing for a thorough examination of regional factors influencing dropout rates.^21^

### Study design and study period of the health demographic survey

The Somaliland Health Demographic Survey (SLHDS) was conducted as a cross-sectional study in 2019 and was subsequently reported as the 2020 SLDHS.

### Sample size and sampling of the health and demographic survey

This study analyzed data from 2741 women aged 15–49 years, collected through the SLHDS 2020, which utilized a stratified, multistage sampling design to ensure representativeness across six geographic zones (Awdal, Woqooyi Galbeed, Togdheer, Sool, Sanaag, and Marodijeex/Sahil). The sample size was determined to provide reliable estimates for maternal health indicators, with enumeration areas selected using probability proportional to size and households chosen systematically within urban, rural, and temporary nomadic settlements (TNS). The SLHDS followed strict ethical protocols, including informed consent and data confidentiality; no additional approval was needed for this secondary analysis of publicly available, de-identified data. To address potential recall bias, the SLHDS minimized recall periods and trained enumerators for accuracy, while stratified sampling mitigated selection bias by ensuring diverse population coverage.

### Variables

#### Outcome variable

The primary focus of this study was the application of ANC among wedded females of reproductive age. The information gathered from the SLDHS was used to assess ANC utilization as a count variable (0, 1, 2, 3, 4, etc.). For this study, the authors classified these options into two categories. Therefore, the outcome variable was measured as a binary outcome. Women who indicated “0” for ANC utilization were considered to have not utilized any ANC, while those who reported “more than 0” were categorized as having utilized ANC. In this study, ANC utilization was defined as attendance at a health facility at least once during the last pregnancy and was considered as utilization of ANC services, while the absence of such attendance was not.

#### Independent variables

Previous investigations have revealed a range of elements connected to the utilization of ANC.^1,9,19,20,22,23^ This study included various independent variables to analyze ANC utilization. Age was grouped into 5-year intervals: 15–19, 20–24, 25–29, 30–34, 35–39, 40–44, and 45–49 years. Respondent’s education level was categorized as no education, primary, secondary, or higher. Employment status for both respondents and their husbands was dichotomized as yes (worked in the last 12 months) or no. Mass media exposure (yes or no) captured access to radio, television, or newspapers. The region was stratified into six zones: Awdal, Woqooyi Galbeed, Togdheer, Sool, Sanaag, and Marodijeex/Sahil. The place of residence was classified as rural, urban, or nomadic. The wealth index was determined using quintiles: lowest, second, middle, fourth, and highest. The total children ever born was categorized as less than 5 or 5 and more, while birth order was divided into firstborn, secondborn, and thirdborn or more. Distance to health facility was a binary variable (yes or no) indicating if distance was a barrier to health care. Finally, pregnancy intention was grouped as “then” (pregnancy wanted at the time), “later” (wanted later), or “no more” (not wanted). These variables provided a comprehensive framework to explore determinants of ANC utilization in Somaliland.

### Data source

The findings of this study were based on the data obtained from the SLHDS. The survey was conducted by trained interviewers using the CSPro Android platform in both the urban and rural regions. In each geographic stratum, 30 homes from each of the 10 enumeration regions were included in the survey, with an additional 30 randomly selected homes from each enumeration region in the nomadic areas. To ensure the accuracy and completeness of the list of homes, the verification process was conducted one day prior to data collection for each TNS.

### Data quality assurance

Before collecting the survey data, training was provided to the data collectors, and a pretest was conducted. In addition, GPS tracking was implemented to aid georeferencing and allow for the collection of geolocated data, and the data collection process was closely monitored.

### Data processing and analysis

Data were obtained from the SLHDS and underwent a thorough cleaning process. Individuals with no outcome variables were removed from the dataset prior to analysis. The data were exported and analyzed using STATA version 17. Descriptive statistics, including mean, frequency, and percentage, were calculated. Bivariate and multivariable binary logistic regression analyses were performed to determine factors associated with ANC. Variables with a *p* < 0.05 were evaluated.

## Results

Table 1 presents the univariate and bivariate analyses of ANC utilization among women in Somaliland using the SLDHS 2020 data. The findings reveal significant variations in ANC utilization based on demographic and socioeconomic factors, as indicated by the *p* values and percentages.

**Table 1.** Univariate and Bivariate Analyses of Antenatal Care Utilization Among Women in Somaliland Using the SLDHS 2020 Data

Variable	Category	Frequency (%)	ANC use during pregnancy		*p*
			Yes (%)	No (%)	
Age in 5-year groups	15–19	165 (6.02)	76 (46.06)	89 (53.94)	0.010
	20–24	538 (19.63)	263 (48.88)	275 (51.12)	
	25–29	757 (27.62)	383 (50.59)	374 (49.41)	
	30–34	557 (20.32)	243 (43.63)	314 (56.37)	
	35–39	460 (16.78)	208 (45.22)	252 (54.78)	
	40–44	200 (7.30)	78 (39.00)	122 (61.00)	
	45–49	64 (2.33)	22 (34.38)	42 (65.63)	
Respondent’s highest education level	No education	2258 (82.38)	928 (41.10)	1330 (58.90)	0.000
	Primary	320 (11.67)	218 (68.13)	102 (31.87)	
	Secondary	109 (3.98)	83 (76.15)	26 (23.85)	
	Higher	54 (1.97)	44 (81.48)	10 (18.52)	
Respondent worked in the last 12 months	Yes	28 (1.02)	20 (71.43)	8 (28.57)	0.008
	No	2713 (98.98)	1253 (46.19)	1460 (53.81)	
Husband ever attended school	Yes	640 (23.35)	445 (69.53)	195 (30.47)	0.000
	No	2101 (76.65)	828 (39.41)	1273 (60.59)	
Husband worked in the last 12 months	Yes	1429 (52.13)	840 (58.78)	589 (41.22)	0.000
	No	1312 (47.87)	433 (33.00)	879 (67.00)	
Mass media exposure	Yes	652 (23.79)	448 (68.71)	204 (31.29)	0.000
	No	2089 (76.21)	825 (39.49)	1264 (60.51)	
Region	Awdal	363 (13.24)	206 (56.75)	157 (43.25)	0.000
	Woqooyi Galbeed	555 (20.25)	196 (35.32)	359 (64.68)	
	Togdheer	589 (21.49)	287 (48.73)	302 (51.27)	
	Sool	607 (22.15)	305 (50.25)	302 (49.75)	
	Sanaag	627 (22.87)	279 (44.50)	348 (55.50)	
Type of place of residence	Rural	844 (30.79)	400 (47.39)	444 (52.61)	0.037
	Urban	847 (30.90)	363 (42.86)	484 (57.14)	
	Nomadic	1050 (38.31)	510 (48.57)	540 (51.43)	
Wealth index	Lowest	713 (26.01)	160 (22.44)	553 (77.56)	0.000
	Second	352 (12.84)	126 (35.80)	226 (64.20)	
	Middle	389 (14.19)	172 (44.22)	217 (55.78)	
	Fourth	568 (20.72)	316 (55.63)	252 (44.37)	
	Highest	719 (26.23)	499 (69.40)	220 (30.60)	
Total children ever born	Less than 5	1486 (54.21)	722 (48.59)	764 (51.41)	0.014
	Five and more	1255 (45.79)	551 (43.90)	704 (56.10)	
Getting medical help for self: distance to health facility	Yes	1785 (65.12)	749 (41.96)	1036 (58.04)	0.000
	No	956 (34.88)	524 (54.81)	432 (45.19)	
Birth order	Firstborn	331 (12.08)	179 (54.08)	152 (45.92)	0.002
	Second	1152 (42.03)	545 (47.31)	607 (52.69)	
	Third or more	1258 (45.90)	549 (43.64)	709 (56.36)	
Wanted pregnancy when became pregnant	Then	1922 (70.12)	952 (49.53)	970 (50.47)	0.000
	Later	686 (25.03)	283 (41.25)	403 (58.75)	
	No more	133 (4.85)	38 (28.57)	95 (71.43)	

SLDHS, Somaliland Demographic Health Survey.

Starting with age, the data shows that among women aged 15–19, 46.06% utilized ANC services, while 53.94% did not, with a *p* value of 0.010 indicating statistical significance. As age increases, ANC utilization rates fluctuate; for instance, women aged 25–29 have the highest utilization at 50.59%. In contrast, those aged 45–49 exhibit the lowest rate at 34.38%. This suggests that younger women may face more barriers to accessing ANC services, emphasizing the need for targeted interventions in this age group.

In terms of education level, the results are striking. Among women with no education, only 41.10% utilized ANC services, while 58.90% did not, with a highly significant *p* value of 0.000. Conversely, the utilization rates increase with educational attainment: 68.13% of women with primary education, 76.15% with secondary education, and 81.48% with higher education utilized ANC services. This demonstrates a clear correlation between educational attainment and health-seeking behavior, highlighting the importance of education in promoting ANC usage.

Employment status also significantly influences ANC utilization. Among women who worked in the last 12 months, 71.43% accessed ANC services, while only 28.57% did not, yielding a *p* value of 0.008. In stark contrast, among those who did not work, only 46.19% utilized ANC, indicating that financial independence may empower women to seek essential health care services.

The educational background of the husband is another critical factor. Among women whose husbands attended school, 69.53% utilized ANC services, compared to 39.41% of those whose husbands did not, with a *p* value of 0.000. This highlights the impact of spousal support and education on women’s health decisions, suggesting that higher education among husbands may positively influence their partners’ access to health care.

Mass media exposure is also correlated with ANC utilization. Women with exposure to mass media had a utilization rate of 68.71%, compared to 39.49% for those without such exposure, and this difference is significant with a *p* value of 0.000. This finding indicates that information dissemination through media channels can significantly enhance awareness and increase health-seeking behaviors.

Geographically, the data reveal disparities in ANC utilization rates across different regions. For example, women in Awdal have a higher utilization rate of 56.75%, while those in Woqooyi Galbeed exhibit a much lower rate of 35.32%, with a significant *p* value of 0.000. This suggests that region-specific factors may hinder access to ANC services, necessitating localized interventions.

Finally, the wealth index illustrates a strong correlation between socioeconomic status and ANC utilization. Among women in the lowest wealth category, only 22.44% utilized ANC services, compared to 69.40% in the highest wealth category, and the *p* value of 0.000 indicates statistical significance. This stark contrast emphasizes the critical influence of economic stability on health care access, suggesting that addressing socioeconomic barriers is essential for improving ANC utilization rates.

Table 2 presents the multivariable logistic regression analysis of ANC utilization among women in Somaliland, utilizing data from the SLDHS 2020. This analysis identifies key factors associated with the likelihood of utilizing ANC services, as indicated by the odds ratios (OR) and corresponding *p* values.

**Table 2.** Multivariable Logistic Regression Analysis of Antenatal Care Utilization Among Women in Somaliland Using SLDHS 2020 Data

Variable	Levels	Odds ratio (OR)	Standard error	Confidence interval (CI)	*p*
Age in 5-year groups	15–19	Reference			
	20–24	1.08	0.22	(0.72, 1.62)	0.695
	25–29	1.23	0.26	(0.81, 1.85)	0.325
	30–34	0.93	0.21	(0.59, 1.45)	0.75
	35–39	1.16	0.28	(0.72, 1.85)	0.524
	40–44	0.99	0.27	(0.57, 1.68)	0.96
	45–49	0.87	0.31	(0.43, 1.74)	0.687
Respondent’s highest education level	No education	Reference			
	Primary	1.50	0.22	(1.13, 1.99)	0.005
	Secondary	1.67	0.42	(1.02, 2.73)	0.041
	Higher	1.89	0.72	(0.89, 3.98)	0.093
Respondent worked in the last 12 months	Yes	Reference			
	No	0.29	0.13	(0.11, 0.68)	0.005
Husband ever attended school	Yes	Reference			
	No	0.64	0.07	(0.51, 0.80)	0.000
Husband worked in the last 12 months	No	Reference			
	Yes	1.40	0.14	(1.16, 1.69)	0.000
Mass media exposure	No	Reference			
	Yes	1.63	0.19	(1.28, 2.04)	0.000
Region	Awdal	Reference			
	Woqooyi Galbeed	0.43	0.07	(0.31, 0.58)	0.000
	Togdheer	0.57	0.09	(0.41, 0.76)	0.000
	Sool	0.62	0.10	(0.45, 0.84)	0.002
	Sanaag	0.48	0.07	(0.35, 0.65)	0.000
Type of place of residence	Rural	Reference			
	Urban	0.85	0.10	(0.68, 1.06)	0.156
	Nomadic	0.52	0.06	(0.41, 0.64)	0.000
Wealth Index	Lowest	Reference			
	Second	1.97	0.31	(1.44, 2.67)	0.000
	Middle	2.69	0.43	(1.96, 3.67)	0.000
	Fourth	3.96	0.62	(2.90, 5.38)	0.000
	Highest	4.70	0.77	(3.40, 6.48)	0.000
Total children ever born	Less than 5	Reference			
	Five and more	2.43	1.34	(0.83, 7.13)	0.105
Getting medical help for self: distance to health facility	Yes	Reference			
	No	1.05	0.10	(0.87, 1.26)	0.623
Birth order	Firstborn	Reference			
	Second	0.86	0.13	(0.63, 1.16)	0.333
	Third or more	0.35	0.20	(0.11, 1.06)	0.065
Wanted pregnancy when became pregnant	Then	Reference			
	Later	0.69	0.07	(0.56, 0.84)	0.000
	No more	0.44	0.09	(0.28, 0.66)	0.000
Constant		3.23	1.71	(1.14, 9.13)	0.027

Beginning with age, the reference group is women aged 15–19, and the OR for older age groups show varying effects. For example, women aged 20–24 have an OR of 1.08 (*p* = 0.695), indicating no significant increase in ANC utilization compared to the reference group. Similarly, the odds ratio for women aged 25–29 is 1.23 (*p* = 0.325), suggesting a slight increase in likelihood, but again, this is not statistically significant. The odds ratios for older age groups, such as 30–34 (OR = 0.93, *p* = 0.750) and 40–44 (OR = 0.99, *p* = 0.960), indicate no meaningful differences in ANC utilization. Overall, age does not appear to be a significant predictor of ANC usage in this analysis.

In contrast, educational attainment is a significant factor influencing ANC utilization. Women with primary education show an OR of 1.50 (*p* = 0.005), indicating a substantial increase in likelihood compared to those with no education. Furthermore, women with secondary education have an OR of 1.67 (*p* = 0.041), reinforcing the positive relationship between education and health-seeking behavior. Although women with higher education exhibit an OR of 1.89, the *p* value of 0.093 suggests this finding is not statistically significant at the conventional level, yet it still hints at a potential positive trend.

The employment status of respondents also plays a crucial role. Women who did not work in the last 12 months have an OR of 0.29 (*p* = 0.005), indicating they are significantly less likely to utilize ANC services compared to their employed counterparts. This finding highlights the importance of financial independence in accessing health care.

The education level of husband’s further influences ANC utilization. Women whose husbands did not attend school have an OR of 0.64 (*p* = 0.000), demonstrating a significant decrease in ANC usage compared to those whose husbands did attend school. This underscores the role of spousal education in promoting health-seeking behaviors among women.

Moreover, mass media exposure significantly impacts ANC utilization, with an OR of 1.63 (*p* = 0.000) for women with media access. This finding suggests that exposure to health information through media channels is crucial in enhancing awareness and encouraging women to seek ANC services.

Geographical disparities are evident in the analysis as well. Women in regions such as Woqooyi Galbeed (OR = 0.43, *p* = 0.000), Togdheer (OR = 0.57, *p* = 0.000), and Sanaag (OR = 0.48, *p* = 0.000) demonstrate significantly lower odds of utilizing ANC services compared to those in the Awdal region. These results highlight the need for targeted interventions in specific regions to improve health care access.

The wealth index also emerges as a significant predictor of ANC utilization. Women in higher wealth categories show progressively higher odds ratios, with the highest wealth category having an OR of 4.70 (*p* = 0.000). This finding clearly indicates that economic status is a major determinant of health care access, emphasizing the importance of addressing socioeconomic barriers in health promotion efforts.

In terms of total children ever born, women with five or more children have an OR of 2.43 (*p* = 0.105), although this result is not statistically significant, it suggests a potential trend worth further investigation. In addition, regarding birth order, the odds ratios for second-born children (OR = 0.86, *p* = 0.333) and third or more (OR = 0.35, *p* = 0.065) indicate no significant differences in ANC utilization.

Finally, the analysis of desired pregnancy reveals that women who wanted their pregnancy at the time of conception have significantly higher odds of utilizing ANC services compared to those who wished for it later (OR = 0.69, *p* = 0.000) or did not want any more children (OR = 0.44, *p* = 0.000). This highlights the importance of pregnancy planning in promoting health-seeking behavior.

**FIG. 1.**
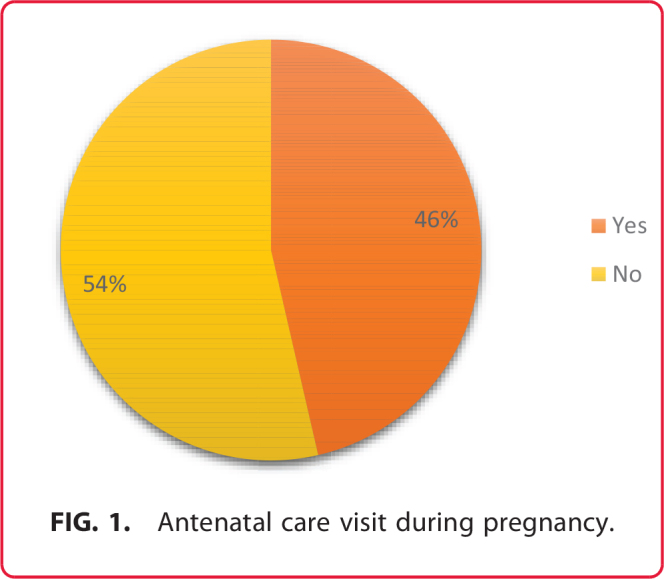
Antenatal care visit during pregnancy.

Figure 1 shows the distribution of respondents regarding their participation in ANC. It revealed that 54% of the respondents did not attend ANC visits during their pregnancy, while 46% did. This indicates that a small majority of the population sampled did not engage in ANC, which could highlight potential barriers to accessing maternal health services or a lack of awareness regarding the importance of such visits for maternal and fetal health. Addressing these barriers and promoting ANC utilization could improve maternal and child health outcomes.

## Discussion

The study aimed to identify the determinants of ANC utilization among reproductive-aged women in Somaliland. The researchers utilized data from the 2020 SLDHS, which included urban, rural, and nomadic residencies across six geographic zones in Somaliland. The study found that higher maternal education levels, urban residence, higher household wealth quintiles, husband’s education level, and exposure to mass media were significantly associated with increased ANC utilization.

The findings of this study are consistent with previous research conducted in various low- and middle-income countries, which have also identified education, economic status, and accessibility as the key determinants of ANC service uptake.^1–3^

Numerous studies have emphasized the importance of maternal education in influencing health-seeking behavior and ANC utilization. For instance, a study in Ethiopia found that women with primary and secondary education had significantly higher odds of utilizing ANC services compared to those with no education.^1^ Similarly, a study in Jordan reported that higher levels of maternal education were associated with increased ANC utilization.^2^

The positive relationship between household wealth and ANC utilization has also been well documented in the literature. A study in India found that women from higher wealth quintiles were more likely to receive the recommended minimum number of ANC visits compared to those from lower wealth quintiles.^10^ Likewise, a study in Niger reported that women’s out-of-pocket costs and time spent accessing ANC services were significant barriers, highlighting the importance of addressing economic factors.^12^

While the study found no significant association between maternal age and ANC utilization, some previous studies have reported varying results. For example, a study in Kenya found that younger women were less likely to attend their first ANC visit in the first trimester, suggesting age-related barriers to early ANC initiation.^14^

In addition, the study did not find a significant relationship between the total number of children ever born and ANC utilization, which contradicts findings from other studies. A study in Ethiopia revealed that women with five or more children had higher odds of utilizing ANC services compared to those with fewer children.^24^

The absence of a significant association between maternal age and ANC utilization in this study contrasts with findings from previous research conducted in other low-resource settings. A potential explanation for this discrepancy is that cultural norms in Somaliland may treat ANC as a universal need across all maternal age groups, rather than varying its importance based on age, as observed in other contexts. In addition, while the total number of children did not significantly predict ANC utilization in this study, this may be due to a saturation effect, where the influence of parity on health-seeking behavior diminishes beyond a certain threshold. Furthermore, methodological differences across studies, including variations in study design, sampling strategies, and measurement techniques, may also contribute to the observed inconsistencies.

Somaliland’s unique sociocultural, geographic, and political contexts profoundly influence maternal health outcomes, including ANC utilization. Traditional gender norms often affect women’s autonomy in health care decision-making, while nomadic lifestyles and dispersed rural populations create geographical barriers to accessing health care facilities. Politically, Somaliland’s unrecognized state status limits international aid and investments, which constrains health care infrastructure development. Understanding these contextual factors is critical for interpreting the determinants of ANC utilization in this region.

Somaliland’s health care system faces significant challenges that limit ANC utilization, including uneven distribution of health care facilities, with urban areas having better access than rural and nomadic regions. A shortage of skilled providers in rural areas results in inconsistent care quality. In addition, financial barriers, compounded by limited government funding and reliance on out-of-pocket payments, restrict access for low-income women. Addressing these issues is critical for improving ANC utilization and maternal health outcomes.

These findings suggest that policymakers should focus on improving health care access in rural and nomadic areas, enhancing education on the importance of ANC, and reducing financial barriers to health care by offering subsidies and supporting infrastructure development.

Strengthens and weaknesses strengths:

- The study utilized a nationally representative dataset (SLDHS 2020), providing robust and generalizable findings.
- The researchers employed appropriate statistical methods, including descriptive and multivariate analyses, to identify the key determinants of ANC utilization.
- The study provides valuable insights for health policymakers and planners in Somaliland to enhance ANC utilization and maternal health outcomes.

Weaknesses:

- The cross-sectional nature of the study limits the ability to establish causal relationships between the identified factors and ANC utilization.
- The study did not explore potential barriers and facilitators to ANC utilization from the perspectives of women and health care providers, which could provide additional contextual understanding.
- The analysis did not delve into the potential interactions and synergistic effects between the various sociodemographic factors, which could offer a more nuanced understanding of the determinants.

Future work plan:

- Conducting a longitudinal study to better understand the dynamic relationships between the identified determinants and changes in ANC utilization over time.
- Incorporating qualitative methods, such as in-depth interviews and focus group discussions, to explore the lived experiences, perceptions, and barriers faced by women in accessing ANC services.
- Examining the potential interactions and synergistic effects between the various sociodemographic factors to uncover more complex pathways influencing ANC utilization.
- Evaluating the quality of ANC services and their impact on utilization and exploring the perspectives of health care providers to identify system-level barriers and facilitators.
- Policymakers should implement educational programs for women and their spouses, alongside providing financial support for economically disadvantaged women. Enhancing health care accessibility in rural and nomadic areas, coupled with mass media campaigns to raise awareness about ANC services, will further improve utilization rates.

## Conclusions

In conclusion, addressing the determinants identified in this study, particularly through improving maternal education, economic support, and health care access, is essential for enhancing ANC utilization in Somaliland. Education plays a crucial role in shaping health-seeking behaviors, and targeted educational interventions aimed at both women and their partners could significantly improve ANC uptake. Economic disparities, as reflected in the differences across wealth quintiles, also highlight the need for policies that reduce financial barriers to accessing maternal health care services. Providing subsidies or financial support for economically disadvantaged women may help bridge this gap.

In addition, improving access to health care in rural and nomadic areas is critical, as geographic disparities can limit ANC utilization. Mass media campaigns could be further leveraged to raise awareness about the importance of early and regular ANC visits, particularly in underserved communities. Policymakers should prioritize a holistic approach that addresses these multifaceted barriers to ensure that all women, regardless of their socioeconomic status or geographic location, have equitable access to ANC services.

Future research should focus on conducting longitudinal studies to better understand the changing dynamics of ANC utilization over time. Incorporating qualitative research methods, such as interviews and focus groups, could also provide deeper insights into the personal and systemic barriers faced by women in accessing maternal care. Finally, evaluating the quality of ANC services and exploring health care provider perspectives will be vital in developing comprehensive strategies to improve maternal health outcomes in Somaliland.

## Data Availability

The dataset used in this study is publicly accessible on the Somalia National Bureau of Statistics website at https://microdata.nbs.gov.so/index.php/catalog/50.
